# In silico bone mechanobiology: modeling a multifaceted biological system

**DOI:** 10.1002/wsbm.1356

**Published:** 2016-09-07

**Authors:** Mario Giorgi, Stefaan W. Verbruggen, Damien Lacroix

**Affiliations:** ^1^ Department of Oncology and Metabolism and INSIGNEO Institute for In Silico MedicineUniversity of SheffieldSheffieldUK; ^2^ Department of BioengineeringImperial College LondonLondonUK; ^3^ INSIGNEO Institute for In Silico Medicine, Department of Mechanical EngineeringUniversity of SheffieldSheffieldUK

## Abstract

Mechanobiology, the study of the influence of mechanical loads on biological processes through signaling to cells, is fundamental to the inherent ability of bone tissue to adapt its structure in response to mechanical stimulation. The immense contribution of computational modeling to the nascent field of bone mechanobiology is indisputable, having aided in the interpretation of experimental findings and identified new avenues of inquiry. Indeed, advances in computational modeling have spurred the development of this field, shedding new light on problems ranging from the mechanical response to loading by individual cells to tissue differentiation during events such as fracture healing. To date, in silico bone mechanobiology has generally taken a reductive approach in attempting to answer discrete biological research questions, with research in the field broadly separated into two streams: (1) mechanoregulation algorithms for predicting mechanobiological changes to bone tissue and (2) models investigating cell mechanobiology. Future models will likely take advantage of advances in computational power and techniques, allowing multiscale and multiphysics modeling to tie the many separate but related biological responses to loading together as part of a larger systems biology approach to shed further light on bone mechanobiology. Finally, although the ever‐increasing complexity of computational mechanobiology models will inevitably move the field toward patient‐specific models in the clinic, the determination of the context in which they can be used safely for clinical purpose will still require an extensive combination of computational and experimental techniques applied to in vitro and in vivo applications. *WIREs Syst Biol Med* 2016, 8:485–505. doi: 10.1002/wsbm.1356

For further resources related to this article, please visit the WIREs website.

## INTRODUCTION

Mechanobiology is a rapidly emerging field of scientific inquiry at the intersection of engineering and biology, and explores the role of mechanical loads in regulating biological processes through signaling to cells. Tissue adaptation in response to changing mechanical loading has been observed in multiple different tissue types and anatomical locations. While new examples of this phenomena are being regularly discovered, such as increased arterial thickness in response to abnormally high blood pressure,^1^ the natural adaptation of bone to mechanical loading has been apparent and studied for over a century.^2^

At its core, mechanobiology is governed by the response of cells within tissue to mechanical forces. It has been shown that most eukaryotic cells exert force on their surrounding tissues even in the absence of any external mechanical stimulus,^3^, ^4^ and that force is essential for basic cellular functions like mitosis and migration.^5^, ^6^ Indeed, it has been proposed that all cells are mechanosensitive.^7^ This occurs through the use of specific molecule or protein complexes known as mechanosensors, broadly organized into three types^3^: (1) attachments between the individual cells, such as stretch‐activated gap junctions, (2) structures on the cell membrane that can deform under fluid flow, such as primary cilia, and (3) attachments between membrane and the extracellular matrix, such as focal adhesions. In each of these cases, a mechanical stimulus is transmitted from the whole‐bone level down to an external cellular feature, and thus into the cytoskeleton or cytoplasm, with the potential to induce a biochemical cascade. This process is known as mechanotransduction, and is the method by which macroscale biological structures and processes can adapt in response to mechanical stimulation.^8^

The study of mechanobiology is particularly significant for bone, which is an adaptive material that employs a complex biological system to remodel itself in response to mechanical stimulation. However, while it is known that these changes are driven by cellular response to loading, it has proven extremely difficult to investigate and predict adaptation in bone experimentally. Thus, researchers have turned to *in silico* modeling techniques in order to elucidate the stimulation these cells experience *in vivo* and to establish mechanobiological rules for these adaptive responses. Advances in computational modeling have spurred the development of this field, shedding new light on problems ranging from the mechanical response to loading by individual cells to predicting tissue differentiation during events such as fracture healing. Therefore, this review will survey the field of *in silico* bone mechanobiology, placing these disparate studies in the broader context of bone mechanobiology and identifying future directions for computational research.

## BONE MECHANOBIOLOGY

Mechanobiology is crucial to the adaptive and regenerative nature of bone. The central question of this field of study is how external muscle loads are transferred to skeletal tissues, how bone cells sense these loads, and how these signals are translated into a cascade of biochemical reactions to produce cell expression or differentiation, ultimately resulting in macroscopic changes to bone structure.^9^ Therefore, bone mechanobiology research must span multiple scales, as bone is a tissue with a complex hierarchical structure that is organized into functional units over multiple dimensions, with the microarchitecture optimized at smaller scales to bear larger scale macroscopic loads.

There is much experimental evidence of bone adapting its mass and structure to different loading conditions, with net bone resorption occurring at low strains and net bone formation occurring at high strains.^10^, ^11^, ^12^, ^13^, ^14^, ^15^, ^16^, ^17^, ^18^, ^19^, ^20^ Moreover, mechanical forces are also known to play a key role in processes such as tissue differentiation^21^ and tissue shape changes.^22^, ^23^, ^24^ For example, axial micro‐interfragmentary movements (micro‐IFMs, small movements in the fragments of bone in a fracture callus) have been shown to reduce the fracture healing time in humans,^25^ and they have also shown mechanical healing improvements by increasing callus stiffness.^26^ In addition, load timings have been shown to be critical for a correct healing process.^27^, ^28^ Indeed, early daily periods of cyclic micro‐IFMs have been shown to improve the healing process,^27^ while an immediate application of loading postsurgery have been shown to have a negative effect on it.^28^ Tissue shape changes have also been linked with mechanical factors. Indeed, it is known that reduced or restricted movements *in utero* increase the risk of bone shape abnormalities in humans. For example, fetal breech position has been shown to increase the risk of hip instability and dysplasia,^29^ ligamentous laxity or malpositioning has been hypothesized to encourage bone deformities,^30^ and studies on the growth plate progression showed abnormal shape development when the angle of the hip joint reaction force changed.^23^, ^31^ Moreover, animal studies have shown abnormal growth under immobilized conditions, further reinforcing evidences that growth and shape changes are dependent on their mechanical environments.^22^, ^24^, ^32^, ^33^, ^34^ Despite the obvious ability of bone to adapt to changing loading conditions, surprisingly little is known about the mechanisms that regulate these changes, and the interplay between them and the manner in which they are stimulated remain to be illuminated.

These adaptive mechanobiological processes are governed by the osteoblasts, osteoclasts, and osteocytes cells working in concert, all capable of transducing mechanical strain signals into biochemical cues for osteogenesis.^35^ At the microscopic scale, most of the cells in the osteogenic lineage [osteoblasts, mesenchymal stem cells (MSCs), osteoclasts, osteoprogenitors, and bone lining cells] are found on the surfaces of bone tissue, and are thus exposed to deformation of the tissue and pressure and fluid flow changes in the surrounding interstitial fluid. Separately, osteocytes are embedded within bone tissue in spaces known as lacunae, which are interconnected by channels known as canaliculi. Macroscopic loading of bone results in strains within the bone matrix, which drives interstitial fluid flow around the lacunar–canalicular network. Therefore, bone cells are exposed to multiple different physical stimuli and at varying magnitudes. Additionally, *in vitro* studies have shown that osteoblastic cells respond with osteogenic signals to both direct matrix strain^36^, ^37^ and to fluid flow *in vitro*.^38^, ^39^, ^40^ This suggests that bone cells can indeed respond to the multiple types of stimulation to which they are exposed *in vivo*. However, it is difficult to determine the magnitude of these stimuli, and how they are transmitted across multiple scales from the whole‐bone level to the level of mechanosensors.

Osteocytes in particular have been shown *in vitro* to be the most mechanosensitive bone cell type, demonstrating a higher intrinsic sensitivity to loading than other osteogenic cells.^41^, ^42^, ^43^ They have also recently been shown to direct osteogenesis in other bone cell types,^44^ reinforcing the theory that osteocytes sense mechanical loading in the bone matrix and then orchestrate the adaptive bone remodeling response.^45^, ^46^, ^47^ Owing to their presence deep within bone matrix, direct experimental observation of osteocytes *in vivo* has proven extremely challenging. As such, the precise mechanical stimuli which they experience *in vivo*, and the mechanisms whereby they sense these stimuli, remain unknown.

The prevalence of *in vitro* culture studies in the field of bone mechanobiology is partly due to the difficulty in experimentally observing mechanosensation by bone cells in their native environment, and is evident in the precious few studies that have investigated these phenomena *in vivo*. High‐resolution two‐dimensional (2D) imaging of lacunae under mechanical loading on an exposed optical microscopy plane demonstrated experimentally that applied strains at the whole‐bone level are amplified in the lacunar matrix.^48^ Separately, as loading‐induced fluid flow is thought to be highly stimulative to osteocytes *in vivo*, fluorescent tracer studies have been performed to examine the fluid flow through the lacunar–canalicular network under mechanical loading.^49^, ^50^, ^51^ Significantly, a recently developed *ex vivo* imaging platform demonstrated intracellular calcium signaling in live osteoblasts and osteocytes, both autonomously and in response to fluid shear mechanical stimulation.^52^, ^53^ Additionally, recently developed techniques have allowed investigation of strain stimulation within *ex vivo* osteocytes on a 2D confocal microscopy plane.^54^

Despite the important *in vitro* and *in vivo* experimental insights outlined above, mechanisms that drive mechanobiological responses, the mechanical environment of bone cells, and the transmission of mechanical loading from higher scales are still poorly understood (Box 1).

BOX 1 **MECHANOBIOLOGY**

Mechanobiology is a nascent interdisciplinary area of research that has recently emerged from the closely related field of traditional biomechanics. While biomechanics is largely concerned with the physical interactions between the body and its surrounding environment, mechanobiology explores the biological responses by tissues and cells when exposed to mechanical stimulation. As mechanobiology involves cell‐driven responses by tissues and organs to loading it is an inherently multiscale field of study, required translation of loading that occurs at the whole‐organ scale down to mechanical stimulation of individual cells. The resulting changes in cell activity are then manifested back up through the scales, causing adaption at the tissue or organ level. Similarly, as biological tissues contain, and are surrounded by, interstitial fluid, mechanical stimulation of cells often comprises a combination of solid and fluid stimulation. Thus, the multiscale and multiphysics nature of mechanobiology provides both challenges and opportunities for this field of frontier science.

## COMPUTATIONAL BONE MECHANOBIOLOGY

**Figure 1:**
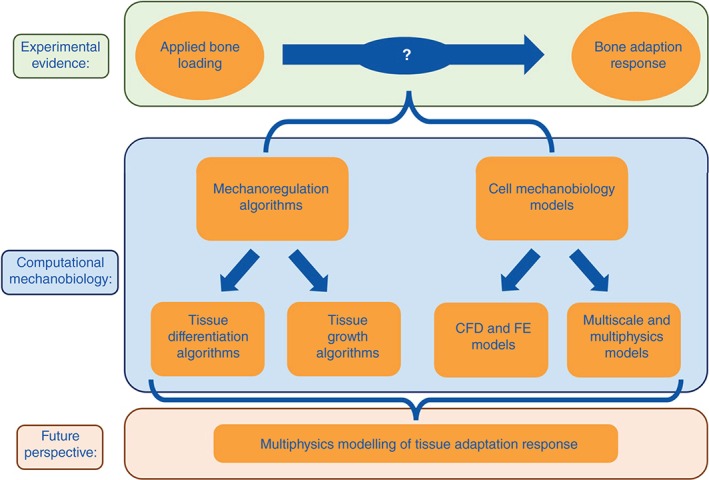
The dichotomy that has developed in computational bone mechanobiology research, as researchers endeavor to understand the adaptive nature of bone.

Computational modeling has grown in prevalence in bone mechanobiology research, and is now recognized as a powerful tool both for probing mechanical interactions at the cellular scale and for predicting important resulting tissue‐level phenomena, such as cell proliferation and differentiation, tissue growth, adaptation, and maintenance. Therefore, research in this field can be broadly separated into two streams (Figure 1): (1) mechanoregulation algorithms for predicting mechanobiological changes to bone tissue and (2) models investigating cell mechanobiology. This dichotomy reflects the current limits of experimental techniques, whereby it is possible to mechanically stimulate bone and quantify the tissue‐level changes that occur, but it is extremely challenging to simultaneously delineate the cellular and molecular mechanisms that give rise to these changes. Computational mechanobiology has endeavored to investigate this gap in our knowledge by either developing increasingly advanced mechanoregulation algorithms to try and predict the observed response to mechanical stimulation, or by modeling individual bone cells to better characterize the manner in which they are stimulated (Box 2).

BOX 2 ***IN SILICO* MODELING**

*In silico* modeling comprises interdisciplinary methods that apply mathematics, physics, and computer science to replicate and analyze the behavior of complex systems through the use of computer simulation. By characterizing a system using numerous variables, the simulation can adjust these variables and predict the resulting effects on the system. *In silico* modeling has developed into a powerful engineering tool to assess the mechanical behavior of physical structures, mechanical systems, and, more recently, biological processes. The primary methods by which this is achieved are finite element (FE) method and finite volume method, whereby the system is broken down into a mesh of smaller, simpler regions, allowing modeling of solid or fluid behaviors, respectively. While FE modeling involves treating these elements like simple structures obeying physical laws, finite volume modeling calculates the change in flow of a fluid through the simple volume and into the next discrete volume. The standard physical equations solved in the elements or volumes are then assembled into a larger system of equations, allowing modeling and analysis of the entire problem.

While these *in silico* methods have provided new insight into both biomechanics and mechanobiology, mechanobiology is an inherently multiscale and multiphysics problem. Therefore, recent advances that have allowed coupling of FE solutions at different scales, and coupling of FE and finite volume simulations through the use of fluid–structure interactions (FSIs), provide fresh opportunities to study mechanobiology and open up new avenues of enquiry.

### Mechanoregulation Algorithms

That mechanical stimulation and bone adaptation are inherently linked has been apparent for centuries, though with discoveries of an ever‐increasing multitude of interrelated physical and biochemical factors influencing bone formation and remodeling, quantitatively predicting tissue response has proven difficult. Initially focused on mechanical stimulation alone, modeling techniques have grown progressively more complex, attempting to bridge the gap between experimentally applied loading and observed response (as summarized in Table 1).

**Table 1:** Material Properties, Applied Mechanical Stimuli, and Resulting Findings From Selected Mechanoregulation Studies

Authors	Application	Material Properties	Stimuli	Outcome
Huiskes et al.^55^	Tissue differentiation	Bone: *E* = 4590, *k* = 3.7e^−13^; fibrous tissue (initial cond.): *E* = 2, *k* = 1.0e^−14^	Fluid/solid velocity, shear strain	Tissue differentiation sequences in agreement with those found experimentally
Lacroix and Prendergast^56^	Tissue differentiation	Granulation tissue: *E* = 0.2, *k* = 1e^−14^, *ν* = 0.167, *S*_bm_ = 2300, *F*_bm_ = 2300, *n* = 0.8; fibrous tissue: *E* = 2, *k* = 1e^−14^, *ν* = 0.167, *S*_bm_ = 2300, *F*_bm_ = 2300, *n* = 0.8; cartilage: *E* = 10, *k* = 5e^−15^, *ν* = 0.167, *S*_bm_ = 3400, *F*_bm_ = 2300, *n* = 0.8; marrow: *E* = 2, *k* = 1e^−14^, *ν* = 0.167, *S*_bm_ = 2300, *F*_bm_ = 2300, *n* = 0.8; immature bone: *E* = 1000, *k* = 1e^−13^, *ν* = 0.3, *S*_bm_ = 13920, *F*_bm_ = 2300, *n* = 0.8; mature bone: *E* = 6000, *k* = 3.7e^−13^, *ν* = 0.3, *S*_bm_ = 13920, *F*_bm_ = 2300, *n* = 0.8; cortical bone: *E* = 20,000, *k* = 1e^−17^, *ν* = 0.3, *S*_bm_ = 13920, *F*_bm_ = 2300, *n* = 0.04	Fluid/solid velocity, shear strain	Cell diffusion rate is a key parameter for healing speed
Geris et al.^57^	Tissue differentiation	Granulation tissue: *E* = 1, *ν* = 0.17, *k* = 1e^−14^; cartilage: *E* = 10, *ν* = 0.17, *k* = 5e^−15^; bone: *E* = 1000, *ν* = 0.3, *k* = 1e^−13^	Fluid/solid velocity, shear strain	Successful prediction of tissue differentiation in a rabbit bone chamber
Heegaard et al.^58^	Joint morphogenesis	Cartilage: *E* = 1.0, *ν* = 0.4; tendons: *E*_L_ = 3.0, *E*_T_ = 0.1, *ν*_L_ = *ν*_T_ = 0.2, *G* = 1.0	Hydrostatic stress	Prediction of congruent surfaces within the joint region
Shefelbine and Carter^59^	Growth front progression	Newly formed bone: *E* = 500, *ν* = 0.2; cartilage: *G* = 2, *ν* = 0.49	Hydrostatic stress, octahedral shear	Successful prediction of normal and abnormal loadings on growth front progression
Isaksson et al.^60^	Bone regeneration	Cortical bone: *E* = 15,750, *k* = 1e^−17^, *ν* = 0.325, *S*_bm_ = 17,660, *F*_bm_ = 2300, *n* = 0.04; marrow: *E* = 2, *k* = 1e^−14^, *ν* = 0.167, *S*_bm_ = 2300, *F*_bm_ = 2300, *n* = 0.8; granulation tissue: *E* = 1, *k* = 1e^−14^, *ν* = 0.167, *S*_bm_ = 2300, *F*_bm_ = 2300, *n* = 0.8; fibrous tissue: *E* = 2, *k* = 1e^−14^, *ν* = 0.167, *S*_bm_ = 2300, *F*_bm_ = 2300, *n* = 0.8; cartilage: *E* = 10, *k* = 5e^−15^, *ν* = 0.167, *S*_bm_ = 3400, *F*_bm_ = 2300, *n* = 0.8; immature bone: *E* = 1000, *k* = 1e^−13^, *ν* = 0.325, *S*_bm_ = 17660, *F*_bm_ = 2300, *n* = 0.8; mature bone: *E* = 6000, *k* = 3.7e^−13^, *ν* = 0.325, *S*_bm_ = 17660, *F*_bm_ = 2300, *n* = 0.8	Fluid/solid velocity, shear strain	Prediction of spatial and temporal tissue distributions observed in distraction osteogenesis experiments
Garcia‐Aznar et al.^61^	Tissue growth/differentiation	Periosteum (initial cond.): *E* = 35.3, *ν* = 0.048; endosteum (initial cond.): *E* = 35.3, *ν* = 0.048; gap: *E* = 8.2, *ν* = 0.048	Second invariant of the deviatoric strain tensor	Correct prediction of callus size in the presence of interfragmentary movements
Giorgi et al.^62^	Joint morphogenesis	Cartilage: *E* = 1.1, *ν* = 0.49; synovial capsule: *E* = 0.287 kPa, *ν* = 0.4	Hydrostatic stress	Prediction of interlocking surfaces for hinge and ball and socket joints
Giorgi et al.^63^	Hip Joint morphogenesis	Cartilage: *E* = 1.1, *ν* = 0.49	Hydrostatic stress	Importance of movements to maintain acetabular depth and femoral head sphericity
Isaksson et al.^64^	Cell and tissue differentiation	Cortical bone: *E* = 15,750, *k* = 1e^−17^, *ν* = 0.325, *S*_bm_ = 17,660, *F*_bm_ = 2300, *n* = 0.04; marrow: *E* = 2, *k* = 1e^−14^, *ν* = 0.167, *S*_bm_ = 2300, *F*_bm_ = 2300, *n* = 0.8; granulation tissue: *E* = 1, *k* = 1e^−14^, *ν* = 0.167, *S*_bm_ = 2300, *F*_bm_ = 2300, *n* = 0.8; fibrous tissue: *E* = 2, *k* = 1e^−14^, *ν* = 0.167, *S*_bm_ = 2300, *F*_bm_ = 2300, *n* = 0.8; cartilage: *E* = 10, *k* = 5e^−15^, *ν* = 0.167, *S*_bm_ = 3400, *F*_bm_ = 2300, *n* = 0.8; immature bone: *E* = 1000, *k* = 1e^−13^, *ν* = 0.325, *S*_bm_ = 17,660, *F*_bm_ = 2300, *n* = 0.8; mature bone: *E* = 6000, *k* = 3.7e^−13^, *ν* = 0.325, *S*_bm_ = 17,660, *F*_bm_ = 2300, *n* = 0.8	Fluid/solid velocity, shear strain	Spatial and temporal predictions of fibrous tissue, cartilage, and bone. Correctly describe fracture healing and disrupted healing
Pérez and Prendergast^65^	Cell and tissue differentiation	Granulation tissue: *E* = 0.2, *k* = 1e^−14^, *ν* = 0.167, *S*_bm_ = 2300, *D* = 0.8; fibrous tissue: *E* = 2, *k* = 1e^−14^, *ν* = 0.167, *S*_bm_ = 2300, *D* = 0.1; cartilage: *E* = 10, *k* = 0.5e^−14^, *ν* = 0.3, *S*_bm_ = 3700, *D* = 0.05; immature bone: *E* = 1000, *k* = 0.1e^−14^, *ν* = 0.3, *S*_bm_ = 13,940, *D* = 0.01; cortical bone: *E* = 17,000, *k* = 0.001e^−14^, *ν* = 0.3, *S*_bm_ = 13,920	Fluid/solid velocity, shear strain	Qualitative agreement with experimental data on bone tissue distribution at the bone–implant interface
Burke and Kelly^66^	Cell differentiation	Granulation tissue: *E* = 0.2, *k* = 1e^−11^, *ν* = 0.167, *μ* = 1e^−9^, *n* = 0.8; fibrous tissue: *E* = 2, *k* = 1e^−11^, *ν* = 0.167, *μ* = 1e^−9^, *n* = 0.8; cartilage: *E* = 10, *k* = 5e^−15^, *ν* = 0.167, *μ* = 1e^−9^, *n* = 0.8; marrow: *E* = 2, *k* = 1e^−14^, *ν* = 0.167, *μ* = 1e^−9^, *n* = 0.8; immature bone: *E* = 1000, *k* = 1e^−13^, *ν* = 0.3, *μ* = 1e^−9^, *n* = 0.8; mature bone: *E* = 6000, *k* = 3.7e^−13^, *ν* = 0.3, *μ* = 1e^−9^, *n* = 0.8; cortical bone: *E* = 20,000, *k* = 1e^−17^, *ν* = 0.3, *μ* = 1e^−9^, *n* = 0.04	Substrate stiffness, oxygen tension	Good agreement with results in fracture repair experiments

*E* is Young's modulus (MPa), *k* is permeability (m^4^/N s), *G* is shear modulus (MPa), *μ* is fluid dynamic viscosity (N s/m^2^), *S*_bm_ is solid bulk modulus (MPa), *F*_bm_ is fluid bulk modulus (MPa), *D* is diffusion coefficient (mm^2^/iter), *n* is porosity, and *υ* is Poisson's ratio.

#### *Tissue Differentiation Algorithms*

Bone, like all musculoskeletal tissues, originates as tissue formed by MSCs and ultimately arises through the process of tissue differentiation. The first theory to propose differentiation into various tissues based on mechanical stimulation was developed by Pauwels,^21^ suggesting that cartilage and bone were promoted by local hydrostatic pressure and shear strain, respectively. This was followed by Perren and Cordey,^67^ who proposed a model based on upper limits of interfragmentary strain to predict fracture gap closure. While this model relied only on axial interfragmentary strains, it predicted that tissue differentiation and associated gap closure would occur if the interfragmentary strain reduced. This resulted in a gradual stiffening of the fracture callus, and they suggested that rigid fixation could slow the onset of fracture healing. Goodship and Kenwright^27^ provided experimental evidence suggesting that interfragmentary motion could accelerate healing, leading to a large body of research attempting to delineate the effects of different types of interfragmentary loading as described in detail elsewhere.^68^

**Figure 2:**
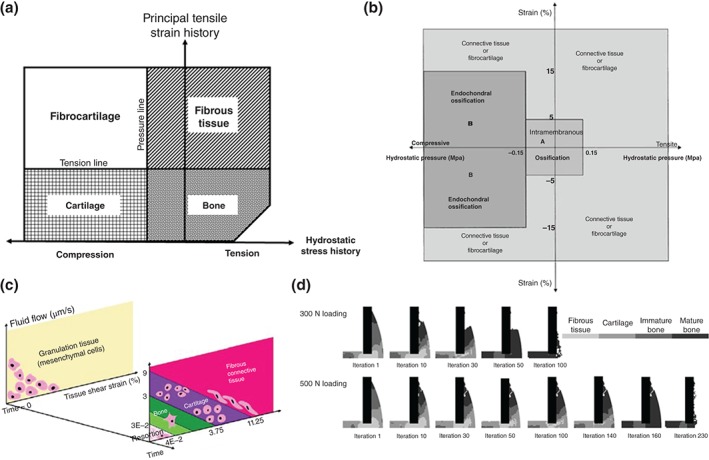
Early tissue differentiation algorithms developed by (a) Carter et al.,^70^ (b) Claes and Heigele,^71^ and (c) Lacroix and Prendergast.^56^ (d) Lacroix et al. implemented their model to predicted healing in a fracture callus.^72^

Based on Pauwel's theory, Carter^69^ introduced a novel approach to understand the influence of cyclic multiaxial stresses on endochondral growth and ossification. In this model, the peak multiaxial stress tensor in a loading cycle was represented by the peak values of two scalar stress invariants, the hydrostatic and octahedral shear stresses. The mechanoregulation algorithm developed suggested that intermittent hydrostatic pressure inhibits growth and ossification of cartilage, while intermittent strain or shear stresses accelerated both processes. By applying this model as a single solid phase using an FE analysis (Figure 2(a)), they investigated fracture healing, as well as joint development and tissue differentiation around implants.^70^

Later, a novel mechanoregulation theory was proposed by Claes and Heigele.^71^ As in the theory proposed by Carter,^69^ this algorithm was based on local strain and hydrostatic pressure to predict pathway of different cellular reactions and tissue differentiation (Figure 2(b)). However, the novelty of this theory relied on an interdisciplinary study where data from animal experiment, cell cultures, and FE analysis were used to evaluate aspects of the process of bone healing.^73^, ^74^ This study allowed them to identify threshold boundaries for the formation of different tissues. Local strains less than 5% in conjunction with values of hydrostatic pressure between ±0.15 MPa were attributed to intramembranous bone formation, while endochondral ossification was promoted by compressive hydrostatic pressure higher than 0.15 MPa and strain lower than 15%.

Given the developing consensus around fluid flow as a stimulus bone cells adaptive response,^42^, ^75^, ^76^ a FE model of a bone–implant interface was developed by Prendergast et al.^55^, ^77^ to explore the influence of mechanical loading on cell differentiation. They showed that the biophysical stimuli experienced by the tissue at the implant interface were not only generated by the tissue matrix but also by the drag force from interstitial fluid flow. Thus, they proposed a biphasic FE model of poroelastic connective tissues comprised of both a fluid and solid phase, where octahedral shear stress and fluid velocity were used as biophysical stimuli, respectively. Lacroix et al. applied this mechanoregulation approach (Figure 2(c)) to investigate tissue differentiation during fracture healing based on a 2D axisymmetric and three‐dimensional (3D) FE model.^56^, ^72^, ^78^ Their adaptive poroelastic model was able to simulate direct periosteal bone formation, endochondral ossification in the external callus (Figure 2(d)), stabilization when bridging of the external callus occurs, and resorption of the external callus.^56^ The model was able to predict slower healing with increasing gap size and increased connective tissue production with increased interfragmentary strain. This model has later been used for successful predictions of tissue differentiation in a rabbit bone chamber^57^ and during osteochondral defect healing.^79^

#### *Tissue Growth/Morphology Algorithms*

**Figure 3:**
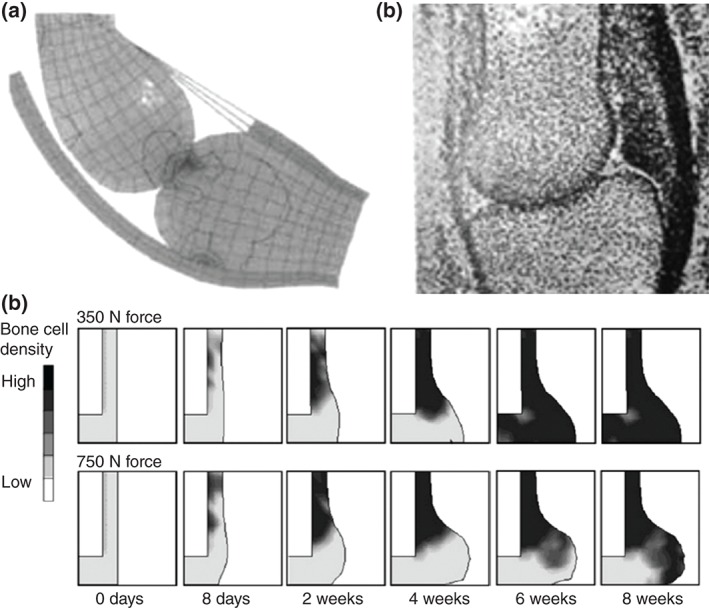
Mechanoregulation models of growth: (a) Prediction of tissue growth in a finger joint developed by Heegaard et al.^58^ provided a good prediction of (b) experimental outcomes.^80^ (c) Growth in a fracture callus under loading predicted by Garcia‐Aznar et al.^61^

While musculoskeletal tissues adapt by differentiation, changes in mass and shape also occur. In spite of this, bone growth in response to mechanical loading has been much less studied. The first computational study to investigate this was developed by Heegaard et al.,^58^ who generated a model to explore how the stresses generated by joint motion may modulate the growth of the cartilaginous rudiments, and lead to the development of a congruent articular surface (Figure 3(a)). They developed a planar biomechanical model of the proximal interphalangeal joint to simulate, using FE analysis, the joint kinematics resulting from muscles contraction, as well as the corresponding stress distribution. Growth, which was predicted by using a variant of the mechanoregulation algorithm proposed by Carter et al.,^69^, ^81^ assumed cyclic hydrostatic compression and tension to inhibit or promote growth, respectively. The model predicted the development of congruent surfaces within the joint region showing consistency with experimental observations^80^ (Figure 3(b)).

Shefelbine and Carter^59^ developed a 3D FE model of the proximal femur to predict the rate of progression of the growth front under normal and abnormal loading conditions. The aim was to link growth front progression with the formation of coxa valga in developmental dysplasia of the hip. The mechanobiological principle used to model growth front changes derived from Carter's theory,^69^, ^81^ where intermittent hydrostatic compressive stresses and intermittent octahedral shear stresses inhibit and promote growth and ossification, respectively. Their simulations predicted a convex growth front shape under assumed normal loading conditions, while growth was promoted on the medial side when abnormal loadings were simulated. The growth front predictions compared well with clinical and histological observation.^82^, ^83^

Another instance in which shape changes as well as tissue changes are known to occur is the fracture callus although, despite the many computational studies of fracture healing described above, most have neglected volumetric growth. By expanding on the algorithm of Prendergast et al.^77^ to include volumetric growth, Isaksson et al.^60^ accurately predicted spatial and temporal tissue distributions observed in distraction osteogenesis experiments. By modeling growth of separate tissue types, it successfully predicted alterations that occurred due to changes in rate and frequency of the distraction. By modeling the matrix production rates of each tissue type using a biphasic swelling model,^84^ growth similar to that observed experimentally was predicted.^60^

A significantly more complex model of callus volumetric growth was proposed by Garcia‐Aznar et al.,^61^ which included cellular parameters such as migration, proliferation, differentiation, and cell death. Variables for the creation and degradation of individual tissues were also incorporated, as well as for tissue damage, calcification, and remodeling. While tissue differentiation was regulated by the second invariant of the deviatoric strain tensor, the volumetric growth was modeled separately using a thermal expansion in FE analysis and controlled according to the amount of tissue production. While anomalies were observed at the boundary conditions of the simulation, increased callus size with interfragmentary movements were correctly predicted^61^ (Figure 3(c)), along with representative changes due to alterations in gap size and fixator stiffness.^85^, ^86^ This model was further adapted by Reina‐Romo et al.^87^ to better account for load history, more successfully simulating distraction osteogenesis. In a subsequent study, they demonstrated that by considering pretraction stresses that arise during distraction osteogenesis, changes in both distraction rate and resulting reaction forces can be accurately predicted.^88^

The most recent study to investigate mechanobiological tissue growth explored how movements and position could impact upon the shape of the developing hip joint,^63^ based on the mechanoregulation algorithm proposed by Giorgi et al.^62^ This algorithm focused on understanding the very early phases of bone development based on the idea that the mechanical stimuli for growth and adaptation of epiphyseal cartilage are different than those that influence endochondral growth and ossification.^62^ They proposed a theory based upon experimental data showing that cyclic hydrostatic compression stimulates matrix production, while static compression inhibits the synthesis of cartilage. When this algorithm was applied to an idealized 2D geometry of a simplified hip joint, they showed that physiological, symmetric movements help to maintain some of the acetabular depth and femoral head sphericity, while reduced or completely absent movements lead to decreased sphericity and acetabular coverage of the femoral head.^63^ The results presented showed consistency with experimental observations.^89^

#### *Modeling of Biological Aspects*

While computational mechanobiology has developed precisely to address the question of tissue adaptation in response to mechanical loading, accounting for the biological factors that mediate this presents a significant challenge. While it is known that cellular proliferation, vascularisation, and nutrient supply are critical for bone regeneration, the mechanisms that link mechanical stimulation to these processes are poorly understood. This is largely due to the difficulty in obtaining *in vivo* experimental data and the resulting reliance upon observations in significantly different *in vitro* conditions, which in turn makes validation of biological assumptions particularly problematic.

**Figure 4:**
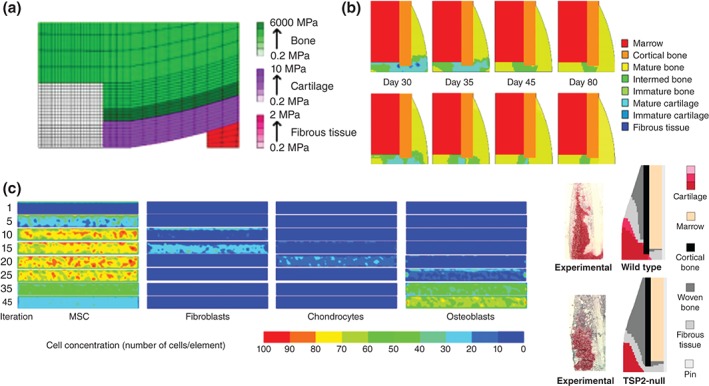
Computational models that take various biochemical factors into account, developed by (a) Kelly and Prendergast,^79^ (b) Isaksson et al.,^64^ (c) Pérez and Prendergast,^65^ and (d) Burke and Kelly,^66^ compared with experimental evidence.^90^

The first attempt to model these factors was made by Lacroix and Prendergast^56^ to model migration, proliferation, and differentiation of cells using a diffusion mechanism. The resulting predictions determined that the cell diffusion rate was the most critical to healing speed. Kelly and Prendergast^79^ developed this model further to include multiple cell phenotypes, allowing for individual elements to represent multiple tissue types (Figure 4(a)). However, the actual rates at which each of these variables occur vary significantly for each tissue that develops during differentiation and healing. While multiple mechanoregulation algorithms have attempted to model bone regeneration (reviewed extensively elsewhere^91^), better characterisation of these rates *in vivo* and incorporation of them into models would likely give greater insight into bone healing rates.^92^

Growth factors, biochemical signals dispatched from cells in response to mechanical stimulation, were chosen as the focus of a mathematical study on fracture healing by Bailon‐Plaza and van der Meulen.^93^ Using a finite difference methodology, cell differentiation was regulated by growth factors, rather than mechanical loading. They used this to quantify changes in cell density, matrix density, and growth factor concentrations, as well as characterizing matrix synthesis and growth factor diffusion.

The first model to link cell phenotype directly to mechanical stimulation was developed by Isaksson et al.,^64^ and included four distinct cell types: MSCs, fibroblasts, chondrocytes, and osteoblasts (Figure 4(b)). Cells were capable of migrating, proliferating, differentiating, or dying, based on both mechanical stimulation and the behavior of adjacent cells. This allowed for spatial and temporal predictions of fibrous tissue, cartilage, and bone, and was shown to correctly describe fracture healing, as well as disrupted healing due to excessive loading or pathology.^64^ For example, alterations due to periosteal stripping or impaired cartilage remodeling (endochondral ossification) compared well with experimental observations.^64^ Parametric data for the study were taken from literature where possible, with a factorial analysis performed to determine key factors and their magnitudes.^94^ Bone healing was predicted to be sensitive to factors involved in fibrous tissue and cartilage formation, with too much or too little soft tissue having a negative effect on the progression of healing.^94^ However, in these studies, all cell activities were modeled on an element basis and anisotropy in the cell movement was not accounted for.

This problem was overcome by Pérez and Prendergast,^65^ who designed a ‘random walk’ model for cell spreading in the callus (Figure 4(c)). This allowed for anisotropic proliferation and migration of cells with a preferential direction. This study built on the mechanoregulation model by Prendergast et al.,^77^ improving on the predictions of that study to include a more irregular distribution of tissue at the bone–implant interface. Comparison of this model to experimental data from a bone chamber showed qualitative agreement,^95^ although the full variability could not be accounted for until individual‐specific cell activity rates were included.^96^ This study demonstrates the importance of identifying cell mechanosensitivity, emphasized by improved predictions compared to clinical outcomes when this model was applied using more realistic 3D loading.^97^

Given the importance of vascular supply to provide nutrients and oxygen to bone cells, some fracture healing models have been based purely on biological factors. A model that accounted for angiogenesis through regulation of cell diffusion and growth factors alone^93^ was further developed to include mechanical stimulation and compared favorably with experimental fracture healing data.^98^, ^99^ Recent developments of these models have focused solely on more realistic representations of angiogenesis.^100^, ^101^, ^102^

Separately, Shefelbine et al.^103^ proposed a modified version of the theory presented by Claes and Heigele^71^ using a 3D μFE model of a fracture gap to simulate trabecular fracture healing, with particular focus on woven bone formation. To determine tissue differentiation over time they used a fuzzy logic controller consisting of a set of 21 linguistic rules. In addition to the mechanical stimuli, the biological factors involved during healing were also included by implementing three changes in the fuzzy rules, each of which represented three different phases of the healing process. With this linear elastic simulation, they were able to simulate the major events of bone regeneration, determining that nutrient supply was the critical factor in bone development and that bone would only form with vascular supply.

Angiogenesis was also the subject of further development of the stochastic cell model by Pérez and Prendergast,^65^ predicting similar capillary networks to those observed experimentally under shear loading. By including mechanical stimulation, they demonstrated that higher loading would slow vascular development resulting in delayed bone formation. The model has also been applied to investigate the discrepancy in bone healing rates between large and small animals,^104^ and has been implemented in a model of a bone tissue engineering scaffold,^105^, ^106^ demonstrating the potential of computational methods to guide regenerative therapies. However, these studies could not account for differences in cell behavior or mechanosensitivity, which are ultimately required to provide a more accurate insight into the cell‐driven mechanobiology of bone.^104^

Most recent developments have modeled the effect of vasculature and loading indirectly, where Burke and Kelly^66^ advanced the methods of Lacroix and Prendergast^56^ to include oxygen tension due to vascular diffusion that occurs in areas where deviatoric strain is lower than 6% (Figure 4(d)). With tissues then differentiating based on the local oxygen tension and the stiffness of the surrounding tissues, good agreement was found with the results in fracture repair experiments.^90^

### Cell Mechanobiology Models

A key limitation of the mechanoregulation algorithms described above is that they remain at the macroscale level of loading and neglect the mechanosensitivity of bone cells themselves.^91^ Modeling cell mechanobiology is particularly challenging owing to the small scales involved and technical limitations of imaging and experimental techniques (as summarized in Table 2). However, this has also provided a window of opportunity for *in silico* modeling to glean new information from experimental observations.

**Table 2:** Material Properties, Applied Mechanical Stimuli, and Resulting Findings From Selected Cell Mechanobiology Studies

Authors	Application	Material Properties	Stimuli	Outcome
Mak et al.^107^	Canalicular flow	Bone (extracellular matrix): *E* = 15,000, *ν* = 0.25, *k* = 0.13e^−15^	2000 με compression	Abrupt changes in drag forces as canaliculus approaches a microporosity (~8e8 Pa/m)
Anderson et al.^108^	Lacunar–canalicular flow	Idealized geometry, *μ* = 0.000855	*P*_i_ = 300 Pa, *P*_o_ = 150, 0 Pa	Cell body primarily exposed to hydrodynamic pressure (~150 Pa), cell processes primarily exposed to shear stress (1.8–7 Pa)
Anderson and Knothe Tate^109^	Lacunar–canalicular flow	Gap size = 0.01–0.2 µm, *μ* = 0.001	Max *V*_i_: 3.28e^−5^ m/s	Physiologically representative localized variations in canalicular geometry increase shear stress stimulation to osteocyte (0.58)
Rath Bonivtch et al.^110^	Lacunar strain	Bone (extracellular matrix): *E* = 25,000, *ν* = 0.3; pericellular matrix: *E* = 15,000–35,000, *ν* = 0.3	2000 με compression	Strain amplification in the lacuna (2957 με), increasing with inclusion of canaliculi (6036 με)
Verbruggen et al.^111^	Osteocyte strain	Realistic geometry (confocal microscopy): bone (extracellular matrix): *E* = 16,000, *ν* = 0.38; pericellular matrix: *E* = 0.04, *ν* = 0.4; osteocyte: *E* = 0.00447, *ν* = 0.3	3000 με compression	Strain amplification in osteocyte due to realistic geometry (24,333 με), and due to ECM projections (12,000 με)
Varga et al.^112^	Osteocyte strain	Realistic geometry (synchrotron X‐ray nano‐tomography): bone (extracellular matrix): *E* = 16,000, *ν* = 0.38; pericellular matrix: *E* = 0.04, *ν* = 0.4; osteocyte: *E* = 0.00447, *ν* = 0.3	1000 με compression	No relationship between morphological parameters and localized strain. Amplification of strain in the lacuna (~10,000 με) and in the osteocyte (~70,000 με)
Verbruggen et al.^113^	Multiphysics osteocyte stimuli	Realistic geometry (confocal microscopy): bone (extracellular matrix): *E* = 16,000, *ν* = 0.38; pericellular matrix: *E* = 0.04, *ν* = 0.4; osteocyte: *E* = 0.00447, *ν* = 0.3; μ = 0.000855	3000 με compression, *P*_i_ = 300 Pa, *P*_o_ = 0 Pa	Multiphysics predictions of interstitial fluid velocity (~60.5 µm/s) and maximum shear stress stimulation (~11 Pa), and osteocyte strain amplification (~10,000 με)
Barreto et al.^114^	Strain stimulation of cytoskeleton	Cytoplasm: *E* = 0.00025, *ν* = 0.49; nucleus: *E* = 0.001, *ν* = 0.3; microtubules: *E* = 2000, *ν* = 0.3; actin cortex: *E* = 0.002, *ν* = 0.3; actin bundles: *E* = 0.341, *ν* = 0.3	0.25 µm compression	Cell stimulation is highly dependent on the thickness, Young's modulus, and rigidity of the actin cortex
Khayyeri et al.^115^	Primary cilia stimulation	Cytoplasm: *E* = 0.00025, *ν* = 0.49; nucleus: *E* = 0.001, *ν* = 0.3; microtubules: *E* = 2000, *ν* = 0.3; actin cortex: *E* = 0.002, *ν* = 0.3; actin bundles: *E* = 0.341, *ν* = 0.3; primary cilia: *E* = 0.178, *ν* = 0.3; *μ* = 0.001	*V*_i_ = 1 mm/s, *V*_o_ = 0 mm/s	Multiphysics model predicts length and stiffness of primary cilium are responsible for transmission of mechanical stimuli to cytoskeleton. Highest strains were found at the base of the primary cilium (~100,000 με)
Vaughan et al.^116^	MSC strain stimulation in bone marrow	Adipocyte: *E* = 0.0009, *ν* = 0.4; MSC: *E* = 0.0025, *ν* = 0.4; plasma: *E* = 0.000001, *ν* = 0.49; trabecular bone: *E* = 10,000, *ν* = 0.3; trabecular bone marrow: *E* = 0.001, *ν* = 0.49	3000 με compression	Osteogenic strain stimulation occurs under normal conditions (~24,000 με), with reduced bone volume fraction leading to increased stimulation (~48,000 με). Increased adipocyte content during osteoporosis reduced MSC stimulation via a shielding effect (~41,000 με)
Vaughan et al.^117^	Multiphysics models of *in vitro* and *in vivo* bone cell mechanosensors	Cytoplasm: *E* = 0.00447, *ν* = 0.4; nucleus: *E* = 0.01788, *ν* = 0.4; primary cilium: *E* = 0.178, *ν* = 0.4; trabecular bone: *E* = 10,000, *ν* = 0.3; trabecular bone marrow: *E* = 0.001, *ν* = 0.49, *μ* = 0.001, *ρ* = 997	*V*_i_ = 34.7 mm/s (*in vitro*), *V*_i_ = 14.8 µm/s (*in vivo*)	Cells highly stimulated *in vitro* by both integrin attachments (>200,000 με) and primary cilium (~220,000 με). *In vivo* cells also highly stimulated by integrin attachments (~270,000 με), while primary cilium was only stimulatory when attached to lacunar bone (~110,000 vs 2000 με)

*E* is Young's modulus (MPa), *k* is permeability (m^4^/N s), *μ* is fluid dynamic viscosity (N s/m^2^), *ρ* is fluid density (kg/m^3^), *υ* is Poisson's ratio, *P*_i_ and *P*_o_ denote pressure at inlet and outlet, and *V*_i_ and *V*_o_ denote velocity at inlet and outlet.

Initially, mathematical models were developed and treated bone as a biphasic continuum, with the application of Biot's poroelastic theory,^118^, ^119^ with these studies asserting that pressure gradients resulting from mechanical loading could generate fluid flow around the osteocyte.^120^ The development of analytical models of idealized osteocyte canaliculi under loading‐induced fluid flow led to predictions of the *in vivo* range for shear stress (0.8–3 Pa) and resulting deformation of osteocyte cell membranes.^121^, ^122^, ^123^, ^124^ In concert with tracer transport experiments,^125^ mathematical models were developed to explain the movement of solute across bone tissue despite cyclic fluid flow under loading,^126^ spawning much debate about the primary mechanisms of loading‐induced fluid flow through the lacunar–canalicular network.^127^, ^128^ Similarly, early attempts to explore strain transfer to the osteocyte cell processes from the surrounding bony matrix applied theoretical modeling techniques.^129^ These models were developed over time to include an internal actin cytoskeleton,^123^ tethering elements anchoring the cytoskeleton to the surrounding matrix,^121^ and discrete focal attachments to projections of the matrix into the canalicular channel.^130^ These models predicted significant amplification of macroscopic strain loads at the cellular level, and reignited debate about whether interstitial fluid flow or bone matrix strain was the primary mechanical stimulus for osteocytes *in vivo*.^131^

#### *Computational Fluid Dynamics and FE Models*

**Figure 5:**
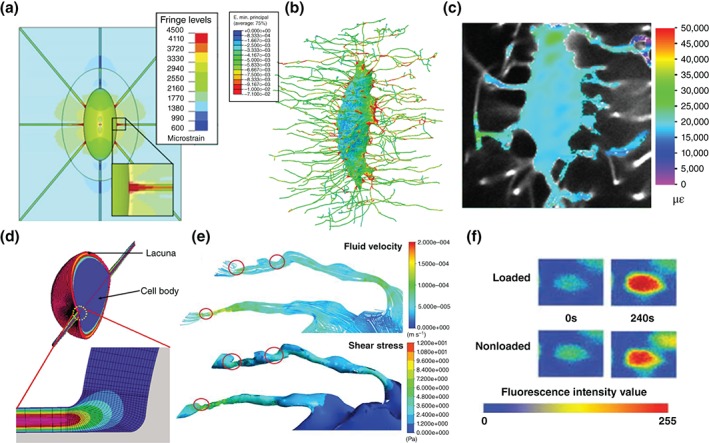
The evolution of finite element (FE) models of bone cells from (a) idealized lacunae^110^ to (b) osteocyte geometries generated from X‐ray nano‐tomography,^112^ predicting strain amplification that has been (c) validated experimentally.^54^ Advances from (d) computational fluid dynamics (CFD) models of osteocytes^108^ with the development of (e) fluid–structure interaction (FSI) techniques,^113^ predicting velocities and shear stresses that have been (f) validated using tracer studies.^51^

Rapid advances in computational power over the past two decades have brought computational modeling to the fore as a key tool to test prevailing theories or develop entirely new ones. Early computational models were developed of an idealized lacunar–canalicular system, predicting abrupt changes in the drag forces within the canaliculi arising from changes in geometry or proximity to bone microporosity and the Haversian canals.^107^ Similar techniques have characterized loading‐induced fluid flow across whole bones.^132^ One study used computational fluid dynamics (CFD) techniques on an idealized model of an osteocyte, predicting high shear stresses within the canaliculi, in contrast to primarily hydrodynamic pressure in the lacunae^108^ (Figure 5(d)). More recently, CFD studies have demonstrated the importance of local geometry on fluid flow in the pericellular space. Models generated 3D approximations of realistic 2D geometries taken from transmission electron microscopy (TEM) images have suggested that localized projections of the bony matrix amplify the fluid shear stimulus to the osteocyte.^109^ Highly detailed 3D geometries of short (80‐nm long) sections of canaliculi have been reconstructed from scans using ultra‐high‐voltage electron microscope, with these models further supporting the theory that 3D geometry greatly affects the velocity of the fluid flow around osteocyte cell processes *in vivo*.^133^ Additionally, numerical models have explored the effect of the pericellular matrix on flow through the canaliculus, investigating the permeability,^134^, ^135^, ^136^ fluid movement,^137^, ^138^ and electro‐chemo‐mechanical effects.^137^, ^139^

In contrast to CFD, the first complete 3D idealized FE model of a whole osteocyte lacuna was developed later, and predicted that strains in the lacunar walls are amplified by a factor of 1.26–1.52 for an applied global strain of 2000 με, increasing to a factor of 3 with the inclusion of canaliculi in the simulations^110^ (Figure 5(a)). While these studies employed idealized geometries, recent FE studies generated accurate 3D geometries of whole osteocytes using confocal laser scanning microscopy, predicting that geometry alone can amplify strain transfer to the osteocyte *in vivo*, both in healthy^111^ and osteoporotic bone.^140^ This was further corroborated by highly detailed FE models generated with geometries of the lacunar–canalicular network captured with synchrotron X‐ray nano‐tomography, which predicted strain amplification of up to a factor of 70^112^ (Figure 5(b)). Some evidence of strain amplification within the lacunar bone matrix under mechanical loading was demonstrated experimentally by Nicolella et al.^48^ However, it was not until very recently that studies validated this computational line of inquiry, using confocal microscopy to directly observe *ex vivo* amplification of applied macroscopic loading when transferred to both osteocytes and osteoblasts live in a rat model^54^ (Figure 5(c)). FE models have also been applied to investigate mechanosensation of bone cells *in vitro*, allowing for direct comparison with cell behavior in a controlled mechanical environment. These have allowed for the exploration of the stimulatory effects of cell morphology, focal attachment density,^141^ and substrate material properties,^142^ as well as the translation of mechanical stimulation to the nucleus via the cytoskeleton.^114^

#### *Multiscale and Multiphysics Models*

Mechanobiology *in vivo* occurs across multiple scales, and so recent studies have applied multiscale modeling techniques alongside periodic boundary conditions to determine that the strain experienced by osteocytes under the same macroscopic loading varies significantly, and strongly depends on their location relative to microstructural porosities.^143^ Furthermore, it was found that lamellar orientation can have a significant effect on strain experienced at the cellular level.^143^ A similar multiscale FE approach has been applied to cells suspended in bone marrow, demonstrating the importance of cell–cell attachments for mechanosensation within the bone marrow under macroscopic bone loading.^116^

As has been discussed, bone cells are exposed to various types of interrelated physical stimuli and therefore reside in a multiphysics environment. Multiphysics modeling is a novel and developing array of methods that couple the effects of several physical phenomena in a single simulation. An example of these are FSI techniques, which couple classic CFD and FE modeling by relaying results between solvers in an iterative manner until a solution to both is converged upon. These new methods have been applied to models of *in vitro* systems, allowing determination of the mechanical stimulation applied to cells by experimental settings,^144^ as well as the stimulation experienced by individual bone cells at different locations in a tissue engineering scaffold.^145^, ^146^ FSI models have also elucidated the function of the primary cilia as a mechanosensor on bone cells, determining the importance of cilia length.^115^ Moreover, researchers have begun to apply FSI to the complex multiphysics environments within bone, recently predicting that stimulatory magnitudes of velocity and shear stress result from macroscopic loading‐induced fluid flow in accurate 3D models of osteocytes^113^ (Figure 5(e)). These results compared favorably with experimental tracer experiments of solute transport in the lacunar–canalicular network^51^ (Figure 5(f)). In an attempt to definitively compare these various mechanosensors, a comprehensive study of bone cell mechanosensation both *in vitro* and *in vivo* used FSI to predict that both integrin attachments and primary cilia are highly stimulated *in vitro*, but that the primary cilia is less stimulated *in vivo* unless embedded in the surrounding matrix.^117^ These multiphysics models in particular demonstrate the value of computational bone cell mechanobiology models for providing information on biophysical parameters that cannot be measured experimentally, as well as the localized effects of multiple types of mechanosensors and complex patterns of physiological loading.

## CONCLUSION AND FUTURE DIRECTIONS

In conclusion, the contribution of *in silico* modeling to the nascent field of bone mechanobiology is indisputable, having aided in the interpretation of experimental findings and identified new avenues of inquiry. The field has progressed from simple 2D models of whole bones with simple material properties to complex 3D models of anatomical bone architecture with more physiological mechanical behavior. Indeed, models have been constructed that can adapt their microarchitecture and mechanical behavior in response to both loading and biological conditions. Similarly, models have been developed of bone at ever smaller scales down to the level of subcellular components. However, despite expanding experimental capabilities, computational modeling has taken a reductive approach in attempting to answer discrete biological research questions. While much has been learned from the notable dichotomy in the computational approaches between mechanoregulation algorithms and cell mechanobiology models, future models must take a more holistic approach to this complex biological system. Advances in computational power are facilitating the development of new computational techniques, allowing coupling of simulations across multiple scales and between multiple physical phenomena.

Indeed, multiscale and multiphysics modeling will play key roles in future efforts to tie the many separate but related biological responses to loading together as part of a larger systems biology approach to shed further light on bone mechanobiology. We have seen early applications of these techniques to mechanobiological problems, and a concerted effort should be made in the next generation of computational models to include information from cell‐level stimulation in mechanobiological algorithms.

These methods will reinforce the initial attempts to apply computational mechanobiology in a therapeutic or clinical setting, in the same manner that computational biomechanics models have aided implant design. Models developed to predict pathological fracture healing have been used to test the spatial and temporal effects of injecting MSCs and growth factors.^147^, ^148^ Moreover, many mechanoregulation algorithms are readily applicable to bone tissue engineering scaffolds, and have aided in the design of various parameters (reviewed in Ref 149). However, one of the key aspects of the development of those algorithms is their validation. They all use different parameters as input or as mechanoregulators and therefore it is difficult to validate each parameter for a specific or a range of clinical applications. The increase in the number of parameters and rules implemented in the algorithms hinder their validation and the ability to falsify them. Therefore, although the ever‐increasing complexity of computational mechanobiology models will inevitably move the field toward patient‐specific models in the clinic, the determination of the context in which they can be used safely for clinical purpose will still require an extensive combination of computational and experimental techniques applied to *in vitro* and *in vivo* applications.
